# Structure and Function of Piezophilic Hyperthermophilic *Pyrococcus yayanosii* pApase

**DOI:** 10.3390/ijms22137159

**Published:** 2021-07-02

**Authors:** Zheng Jin, Weiwei Wang, Xuegong Li, Huan Zhou, Gangshun Yi, Qisheng Wang, Feng Yu, Xiang Xiao, Xipeng Liu

**Affiliations:** 1 State Key Laboratory of Microbial Metabolism, School of Life Sciences and Biotechnology, Shanghai Jiao Tong University, 800 Dong-Chuan Road, Shanghai 200240, China; jinzheng_sjtu@163.com (Z.J.); 13166228531@163.com (G.Y.); 2 Shanghai Institute of Applied Physics, Chinese Academy of Sciences, No. 239 Zhangheng Road, Shanghai 201204, China; wangweiwei@sinap.ac.cn (W.W.); zhouhuan@sinap.ac.cn (H.Z.); wangqisheng@zjlab.org.cn (Q.W.); 3 Institute of Deep-Sea Science and Engineering, Chinese Academy of Sciences, No. 28 Luhuitou Road, Sanya 572000, China; xuegongli@idsse.ac.cn; 4 Joint International Research Laboratory of Metabolic & Developmental Sciences (Ministry of Education), Shanghai Jiao Tong University, 800 Dong-Chuan Road, Shanghai 200240, China; 5 State Key Laboratory of Ocean Engineering, School of Naval Architecture, Ocean and Civil Engineering, Shanghai Jiao Tong University, 800 Dong-Chuan Road, Shanghai 200240, China; 6 Southern Marine Science and Engineering Guangdong Laboratory (Zhuhai), Zhuhai 519080, China

**Keywords:** *Pyrococcus yayanosii*, pAp, pApase, DHH phosphoesterase

## Abstract

3’-Phosphoadenosine 5’-monophosphate (pAp) is a byproduct of sulfate assimilation and coenzyme A metabolism. pAp can inhibit the activity of 3′-phosphoadenosine 5′-phosphosulfate (PAPS) reductase and sulfotransferase and regulate gene expression under stress conditions by inhibiting XRN family of exoribonucleases. In metazoans, plants, yeast, and some bacteria, pAp can be converted into 5’-adenosine monophosphate (AMP) and inorganic phosphate by CysQ. In some bacteria and archaea, nanoRNases (Nrn) from the Asp-His-His (DHH) phosphoesterase superfamily are responsible for recycling pAp. In addition, histidinol phosphatase from the amidohydrolase superfamily can hydrolyze pAp. The bacterial enzymes for pAp turnover and their catalysis mechanism have been well studied, but these processes remain unclear in archaea. *Pyrococcus yayanosii*, an obligate piezophilic hyperthermophilic archaea, encodes a DHH family pApase homolog (PyapApase). Biochemical characterization showed that PyapApase can efficiently convert pAp into AMP and phosphate. The resolved crystal structure of apo-PyapApase is similar to that of bacterial nanoRNaseA (NrnA), but they are slightly different in the α-helix linker connecting the DHH and Asp-His-His associated 1 (DHHA1) domains. The longer α-helix of PyapApase leads to a narrower substrate-binding cleft between the DHH and DHHA1 domains than what is observed in bacterial NrnA. Through mutation analysis of conserved amino acid residues involved in coordinating metal ion and binding substrate pAp, it was confirmed that PyapApase has an ion coordination pattern similar to that of NrnA and slightly different substrate binding patterns. The results provide combined structural and functional insight into the enzymatic turnover of pAp, implying the potential function of sulfate assimilation in hyperthermophilic cells.

## 1. Introduction

Sulfate assimilation is an important biological process that exists in metazoans, plants, fungi, and some prokaryotes [1,2,3,4]. It can produce sulfur-containing amino acids, derivatives of sulfur-containing amino acids, and sulfonated products through 3′-phosphoadenosine 5′-phosphosulfate (PAPS). In sulfate assimilation, PAPS is used as a sulfate donor by PAPS reductase to generate sulfite or by sulfotransferase to produce sulfonated molecules and proteins [5,6,7,8], accompanied by the generation of 3′-phosphoadenosine 5′-monophosphate (pAp). As a byproduct in PAPS metabolism, pAp plays important roles in cell functions. It can inhibit the activities of PAPS reductase and sulfotransferase in sulfate assimilation [9] and the activity of oligoribonuclease (Orn) during oligo RNA degradation [10], and regulate gene expression by inhibiting the XRN family of exoribonucleases in plants [11,12].

pAp is hydrolyzed by specialized phosphoesterases to generate 5’-adenosine monophosphate (AMP) and inorganic phosphate (Pi). These specialized phosphoesterases include CysQ from the FIG superfamily (fructose-1,6-bisphosphatase/inositol monophosphatase/glpX), nanoRNases (Nrn) from the Asp-His-His (DHH) phosphoesterase superfamily, and histidinol phosphatase from the amidohydrolase superfamily. In metazoans, plants, yeast, and some bacteria, pAp is hydrolyzed by CysQ homologous proteins [13,14,15]. In many bacteria lacking the *cysQ* gene, pAp is hydrolyzed by members of the DHH phosphoesterase superfamily, such as NrnA and RecJ-like nucleases [16,17]. Cv1693, a histidinol phosphatase from *Chromobacterium violaceum*, belongs to the amidohydrolase superfamily and can also hydrolyze pAp [18].

DHH superfamily phosphoesterases contain both DHH domain and Asp-His-His associated (DHHA) domain. The DHH domain contains several conserved acidic residues to coordinate two divalent cations that in turn activate a water molecule for nucleophilic attack on the phosphodiester backbone [19]. These cation coordination residues belong to four motifs (I–IV) located in the N-terminal catalytic domain. Among the four motifs, motif III consists of three tandem DHH conserved residues and is the nomination origin of this superfamily. DHH superfamily members harbor different DHHA domains for substrate binding in the C-terminal domain. According to the residues of the DHHA motif, the DHH superfamily is classified into the DHHA1 and DHHA2 families.

The DHHA1 family possesses a typical conserved motif and contains many members, including the nucleases RecJ [20], NrnA [16], and Hef-associated nuclease (HAN) [21]. In bacteria, the NrnA subfamily shows strong phosphoesterase activity on the 3′ phosphoester bond of pAp. In addition to pApase activity, NrnA also shows activity on other substrates. NrnA from Bacillus subtilis (BsNrnA) can rescue the phenotype of Escherichia coli (*E. coli*) deficient in both *orn* and *cysQ*, indicating that it can degrade not only nanoRNA (2–5 nt) but also pAp [16]. BsNrnA can also hydrolyze longer RNA substrates from the 5′ end at a very low rate [22]. In *Mycobacterium tuberculosis* (*M. tuberculosis*), NrnA can convert nanoRNA, pNpN, pAp, and c-di-AMP into nucleotides [23,24]. In addition to pAp, the RecJ-like protein TTHA0118 from *Thermus thermophilus (T. thermophilus)* HB8 can hydrolyze ssDNA, ssRNA, cAMP, and cGMP at low rates [17]. It is inferred that the interaction between the DHH and DHHA1 domains results in various substrate accommodation mechanisms, which are the structural basis for DHHA1 family members to hydrolyze different substrates.

There are many reports on pAp hydrolase in bacteria and eukaryotes [13,16,25,26]. However, the turnover pathways of pAp and key enzyme(s) are less well studied in archaea. Although it has been reported that both the RecJ family protein Ton_1706 in *Thermococcus onnurineus* (*T. onnurineus*) NA1 [27] and the NrnB family protein APE_0124 in *Aeropyrum pernix* (*A. pernix*) [28] can convert pAp into AMP and Pi, the detailed enzymatic properties and catalytic mechanism have not been clarified for archaeal pApases. To understand the function of archaeal pApase in detail, the NrnA homolog PYCH_17540 from *Pyrococcus yayanosii* (*P. yayanosii*) CH1 was chosen as a candidate, and its potential role in pAp turnover was characterized. Enzymatic kinetics show that the catalytic constant k_cat_ of pApase from *P. yayanosii* (PyapApase) is much higher than that of *E. coli* CysQ and BsNrnA NrnA [16], although the k_cat_/K_m_ value is almost equal to *E. coli* CysQ [27]. Crystallographic studies show that the structure of apo-PyapApase is similar to that of NrnA [22]. Both of them contain a DHH domain and DHHA1 domain linked by an α-helix. However, the α-helix of PyapApase is longer than that of bacterial NrnA. As a result, PyapApase forms a narrower substrate-binding cleft between the DHH and DHHA1 domains and shows higher substrate specificity than bacterial NrnA. Based on its crystal structure, the ion coordination pattern of PyapApase was confirmed to be similar to that of bacterial NrnA family pApase by analyzing the enzymatic activity of site-directed mutants of conserved residues involved in coordinating metal ion. These results provide structural and functional insight into the enzymatic turnover of pAp, implying the existence of a functional pathway of sulfate assimilation and/or nanoRNA recycling in hyperthermophilic cells.

## 2. Results

### 2.1. The Gene Cluster for Assimilatory Sulfate Reduction

**Figure 1:**
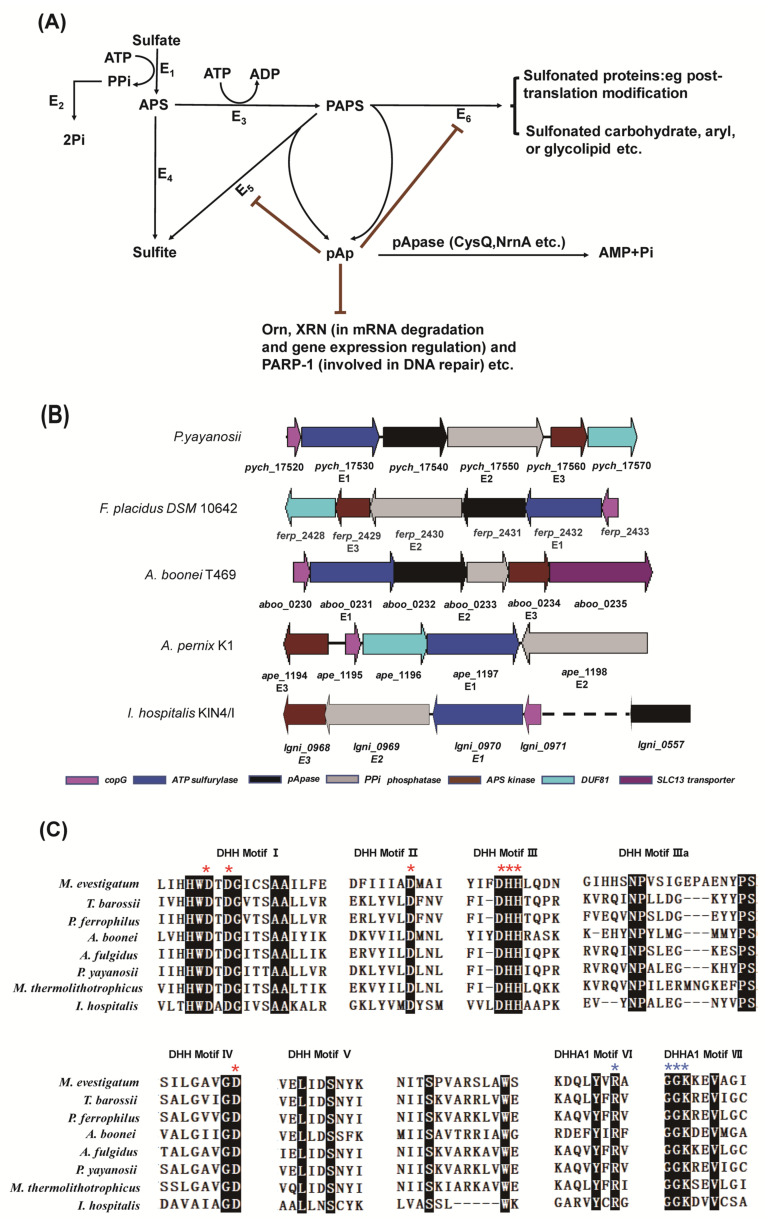
Archaeal sulfate assimilation pathway and function of pApase. (**A**) Archaeal sulfate assimilation pathway candidate in cells. Enzymes are shown alongside the black arrows (E1: ATP sulfurylase; E2: PPi phosphatase; E3: APS kinase; E4: APS reductase; E5: PAPS reductase; and E6: sulfotransferase), and the brown lines indicate inhibition by pAp. (**B**) Conserved gene cluster involved in the sulfate assimilation pathway and auxiliary genes in Euryarchaeota and Crenarchaeota. Information for strains and related genes are as follows: *P. yayanosii* CH1 (*pych_17520* to *17570*), *Ferroglobus placidus* DSM 10642 (*ferp_2428* to *2433*), *Aciduliprofundum boonei* T469 (*aboo*_*0230* to *0235*), *A. pernix* K1 (*ape*_*1194.1* to *1198.1*) and *Ignicoccus hospitalis* KIN4/I (*igni*_*0968* to *0971* and *igni*_*0557*). (**C**) Multi-alignment of the conserved motifs of pApases from different archaeal strains, including *Methanohalobium evestigatum* Z-7303 (Metev_1146), *Thermococcus barossii* (A3L01_09430), *Palaeococcus ferrophilus* (WP_048150725.1), *Aciduliprofundum boonei* T469 (Aboo_0232), *Methanothermococcus thermolithotrophicus* (WP_018153796.1) and *P. yayanosii* CH1 (PYCH_17540), *Archaeoglobus fulgidus* (AF_0291), and *Ignicoccus hospitalis* KIN4/I (Igni_0557). The alignment was performed using ClustalW, and the completely conserved residues are shaded in dark. The mutated conserved residues for coordinating metal ions (D10, D12 D59, D77, H78, H79, and D126) and binding substrates (R259, G285, G286, and R287) are indicated with red and blue asterisks, respectively, above the PyapApase sequence.

A gene cluster involved in assimilatory sulfate reduction exists in the genome of some strains from Euryarchaeota and Crenarchaeota, including *T. onnurineus* NA1 [27] and *P.* *yayanosii* (Figure 1A). The intact gene cluster consists of five genes, *pych_17520* to *pych_17560*, in *P. yayanosii* (Figure 1B). PYCH_17520 is a putative copG transcription factor and potentially regulates the pathway of assimilatory sulfate reduction. PYCH_17530 and PYCH_17560 are ATP sulfurylase and APS kinase, respectively, and work together to generate the PAPS. PYCH_17540 and PYCH_17550 are annotated as DHH phosphoesterase and type I phosphodiesterase/nucleotide pyrophosphatase, respectively. It is speculated that PYCH_17540 (pApase) degrades pAp and pApS, and PYCH_17550 degrades PPi, the byproduct of PAPS synthesis.

The typical homologs of PYCH_17540 were selected from different taxa to analyze the conserved motifs. These homologs have seven conserved motifs (Figure 1C) and are annotated as members of a type of pApase in archaea. A phylogenetic tree of pApase was constructed using MEGA X [29] with 1000 cycles of bootstrap for selected sequences (Figure S1), showing that the pApases form a separate branch and are derived from the common ancestor with NrnA and RecJ nucleases.

### 2.2. PyapApase Can Hydrolyze pAp In Vitro

**Figure 2:**
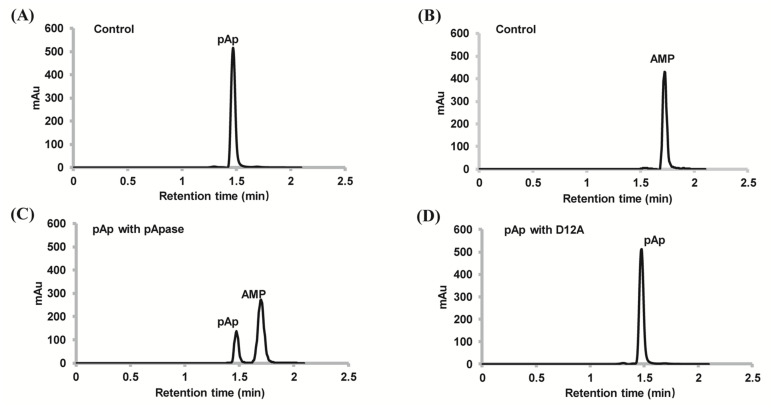
pApase degrades pAp into 5′AMP and PPi. Standard elution curves of pAp (**A**) and AMP (**B**). Hydrolysis of pAp by wt pApase (**C**) and ion binding deficient mutant D12A (**D**). Additionally, 0.2 mM pAp was incubated with 3.75 nM of PyapApase or its mutant D12A at 70 °C for 5 min in reaction buffer consisting of 20 mM Tris-HCl pH 8.0, 50 mM NaCl, and 0.5 mM Mn^2+^.

The homolog of PyapApase (PYCH_17540) in *T. onnurineus* NA1 has been reported to degrade pAp and PAPS [27]; therefore, the ability of PyapApase to hydrolyze pAp was first characterized. Wild-type (wt) PyapApase could efficiently degrade pAp and produce 5′-AMP and Pi, whereas the inactive mutant D12A could not (Figure 2).

### 2.3. The Optimal Reaction Conditions

**Figure 3:**
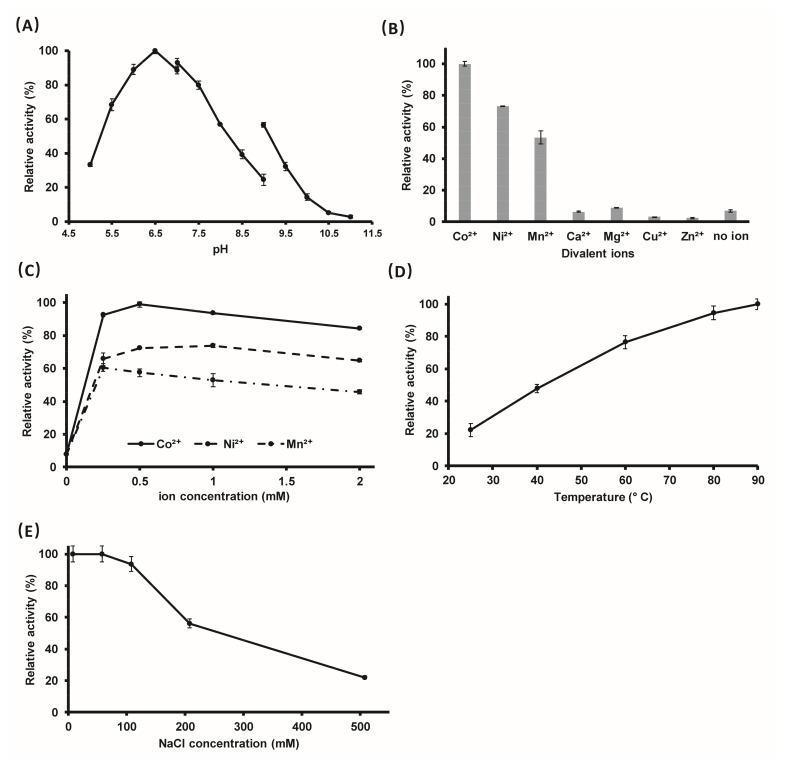
Effect of pH (**A**), divalent metal ions (**B**), divalent metal ion concentrations (**C**), NaCl concentrations (**D**), and reaction temperature (**E**) on the activity of PyapApase. Each reaction contains 0.2 mM of pApase and 3.75 nM of pAp. (**A**) pH-dependent reactions were run with the following buffers (each at 20 mM): MES-NaOH (pH 5–7), Tris-HCl (pH 7–9), and Gly-NaOH (pH 9–11). (**B**) Divalent metal ions were present at a concentration of 1 mM. (**C**) Divalent metal ion (Ni^2+^, Co^2+^, Mn^2+^) dependence over a concentration range of 0 to 2 mM. (**D**) NaCl concentration over a range of 15 to 500 mM. (**E**) Temperature dependence over a range of 25 to 90 °C. Activities are expressed as percentages of the best condition for each parameter: pH 6.5 (**A**), Co^2+^ (**B**), 0.5 mM Co^2+^ (**C**), 50 mM NaCl (**D**), and 90 °C (**E**).

The above results show that PyapApase possesses the intrinsic activity of hydrolyzing pAp into AMP and phosphate. Then, the optimal reaction conditions for hydrolyzing pAp were determined in vitro. The effects of pH value, divalent metal ions, and their concentration, temperature, and NaCl concentration on enzymatic activity were characterized. PyapApase hydrolyzes pAp with higher efficiency in buffers with pH values between 5.5 and 8.0 and with an optimal pH value of 6.5 (Figure 3A). For seven divalent metal ions that were supplemented, Co^2+^ is the optimal cofactor of PyapApase, followed by Ni^2+^ and Mn^2+^, with which the enzyme showed 73% and 53% activity relative to Co^2+^, respectively. Although PyapApase could hydrolyze pAp at a lower efficiency without adding metal divalent ions, it was completely inactivated in the presence of EDTA, indicating that divalent ions are essential for activity and that part of the purified PyapApase bound divalent ions during recombinant expression in *E. coli* cells or bound Ni^2+^ during Ni^2+^ affinity column purification. However, Ca^2+^, Cu^2+^, and Zn^2+^ inhibited PyapApase and Mg^2+^ did not show clear effects (Figure 3B). The optimum concentrations for Ni^2+^, Co^2+^, and Mn^2+^ are 0.5, 0.5, and 0.25 mM, respectively. (Figure 3C). The reaction rate of PyapApase increases continuously with increasing temperature from 25 to 90 °C, and the enzyme activity at 90 °C is almost four times higher than that at 25 °C (Figure 3D), consistent with the fact that PyapApase is an enzyme from hyperthermophiles. PyapApase shows higher activity below 100 mM NaCl, and more than 100 mM NaCl inhibits its activity (Figure 3E). The enzyme activity at 50 mM NaCl was five-fold higher than that at 500 mM NaCl.

### 2.4. Substrate Specificity of PyapApase Is High

The substrates of the reported bacterial DHH family pAp hydrolases are broad, such as TTHA0118 from *T. thermophilus* and Rv2837 from *M. tuberculosis* [17,23,24]. The substate specificity of PyapApase was determined. In contrast to bacterial pApase, archaeal PyapApase did not show the ability to hydrolyze cyclic and linear dinucleotides such as c-di-AMP, c-di-GMP, pApA, and pGpG (Figure S2). Similar to the results with cyclic and linear nucleotides, single-stranded (ss) RNA and ssDNA are also not catalytic substrates of PyapApase (Figure S3). Even with 1000 times greater concentrations than the pAp hydrolysis reaction, pApase cannot hydrolyze ssDNA and ssRNA. However, at much lower enzymatic concentrations, bacterial Rv2837c [23,24] and archaeal Ape_0124 [28] can efficiently hydrolyze cyclic nucleotides, nanoRNAs, and short ssDNA. These results confirm that PyapApase has a high substrate specificity.

### 2.5. PyapApase Possesses a Similar Structure to Bacterial pApase

**Figure 4:**
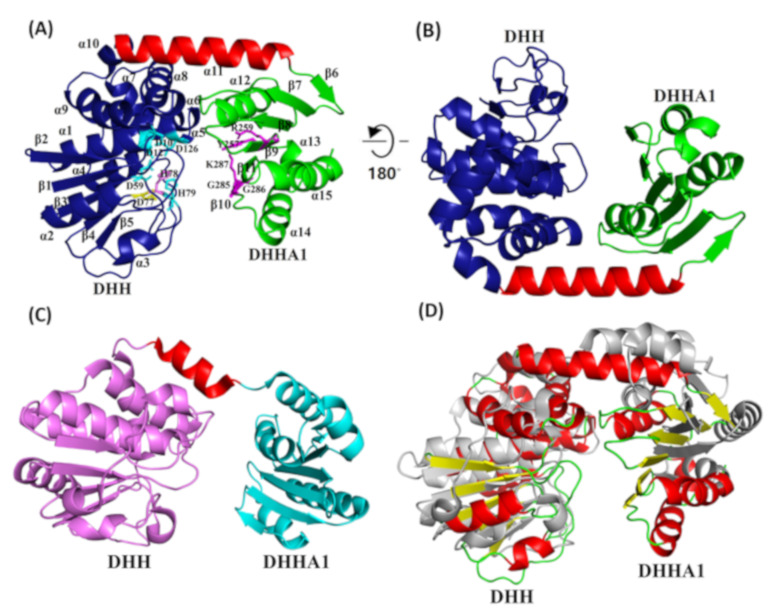
The overall crystal structures of archaeal PyapApase and its bacterial homologs. The overall crystal structure of archaeal PyapApase (**A**), rotation of 180° (**C**), and bacterial BsNrnA ((**B**), PDB ID: 5J21) are shown as cartoon models. The DHH domain of these proteins is colored blue, α-helix linkers are red, and the DHHA1 domain is green. (**D**) The 3D superimposition of PyapApase (colored) and bacterial NrnA (gray, 5J21). The conserved key residues involved in coordinating divalent metal ions (DHH domain) and binding substrates (DHHA1 domain) are shown in ball-and-stick form.

The crystal structures of wt and site-mutated PyapApase were resolved (Table 1). The crystal structure of wt PyapApase has the typical structure of the DHH phosphoesterase superfamily (Figure 4). The overall structure is composed of a DHH domain and a DHHA1 domain connected by a longer α-helix (Figure 4A,B). The DHH domain is mainly composed of seven α-helixes and five β-sheets, and the DHHA1 domain is composed of four α-helixes and six β-sheets. The cleft between the DHH and DHHA1 domains is the substrate binding cleft and active site. All conserved residues (shown in Figure 1C) predicted to participate in divalent ion coordination or substrate binding are marked in the crystal structure (Figure 4A). Based on the crystal structure of BsNrnA, the two catalytic divalent metal ions are predicted to be coordinated by four residues, D10 and D12 in motif I, D59 in motif II, H78 in motif III, and D126 in motif IV, located at the active center. The substrate is predicted to be bound by R259 in motif VI and G285 and K287 in motif VII.

However, compared with bacterial NrnA (Figure 4C), the linker α-helix of PyapApase is much longer than that of NrnA, which possesses a shorter α-helix adjacent to the disordered region (Figure 4D). As a result, PyapApase has a narrower substrate-binding cleft between the DHH and DHHA1 domains than bacterial NrnA. Meanwhile, the mutated DHH motif did not clearly change the three-dimensional structure (Figure S4). However, the mutant had a clearly different active site compared with the wt enzyme, and the altered residues lost the ability to coordinate divalent metal ions.

### 2.6. Functions of Key Amino Acid Residues

**Figure 5:**
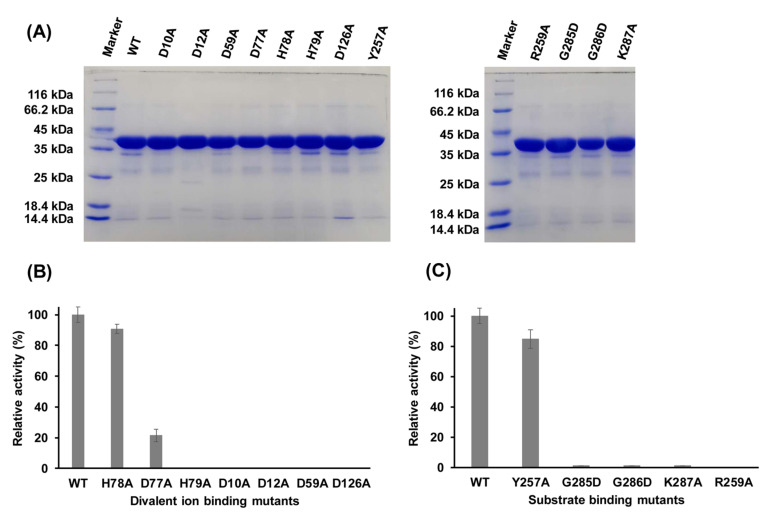
Verification of active sites of PyapApase. (**A**) Purity of wt and mutated pApases confirmed by 12% SDS-PAGE. (**B**) Relative activity of mutants involved in divalent ion binding sites. (**C**) Relative activity of mutants involved in substrate binding. Activities are expressed as percentages of the activity of wild-type pApase. In each reaction, 0.2 mM of pAp was incubated with 3.75 nM of purified proteins at 70 °C for 2 min.

In several DHH superfamily members, such as bacterial NrnA and RecJ, motifs I–IV are involved in coordinating a divalent metal cofactor that is necessary for hydrolyzing the phosphodiester bond [20,21,22]. In PyapApase, residues D10 and D12 in motif I, D59 in motif II, H78 in motif III, and D126 in motif IV (Figure 1C and Figure 4A and Figure S5) are predicted to be mainly responsible for binding Mn^2+^ based on the superimposition of the structures of PyapApase and BsNrnA. Mutation of these conserved key residues resulted in inactivation of nuclease activity (Figure 5), suggesting that these conserved residues are involved in coordinating Mn^2+^, the PyapApase cofactor. Based on the structure of PyapApase and other members of the DHH superfamily, H78 in motif III should be involved in ion coordination, but H78A has almost no effect on PyapApase activity. The D77A and H79A mutants in motif III lost more than 80% and 100% activity. These results for the DHH motif are consistent with the structure of PyapApase with a mutated DHH motif (Figure S4).

The modeled complex structure of BsNrnA with pAp [22] showed that several conserved residues are responsible for substrate binding (Figure S5). For binding pAp, R262, R264, and R266 in motif VI are responsible for binding the nonesterified oxygen atoms of the 5′ phosphate of pAp by its side chain guanido groups, G282 for binding the adenosine base via carbonyl oxygen, and H284 for contacting the phosphodiester backbone toward the 3′ end of the substrate [22]. In PyapApase, the residues corresponding to R266, G282, and H284 are R259, G285, and K287, respectively. All mutants of R259A, G285D, and K287A inactivated pApase activity (Figure 5), indicating that these residues are required for substrate binding.

Mutants of other conserved residues that may be involved in substrate binding were also constructed to determine their effects on enzyme activity. G286 is the third G residue in motif VII of GGGH. The results show that G286D is inactive (Figure 5), suggesting that G286 may have an effect on substrate binding. Y257 is a conserved residue in motif VI of archaeal pApase, but it is not conserved in motif VI of other DHH superfamily members. Mutant Y257A had little effect on enzyme activity (Figure 5), indicating that Y257 may not be involved in substrate binding.

### 2.7. Enzyme Kinetics

The kinetic constants of the wild type, putative divalent metal ion-binding mutants, and substrate binding mutants were measured to further determine the catalytic efficiency of PyapApase and the functions of these mutated residues. The K_m_ and k_cat_ of wild-type PyapApase were 272 μM and 1259 s^−1^, respectively. The k_cat_/K_m_ value of PyapApase is 4.6 × 10^6^ M^−1^s^−1^, approximately equal to that of other reported pAp hydrolases, such as CysQ in *E. coli* and pApase in mammal [27,30].

The metal ion-binding mutant D12A has kinetic values of K_m_ and k_cat_ of 790 μM and 1.71 s^−1^, respectively. The largely decreased k_cat_, approximately 1/1000 of the wild type, indicates that this mutant mainly affects the catalytic step, consistent with the role of D12 as a metal ion coordination residue in the catalytic center.

The substrate binding mutants R259A in motif VI and G285D in motif VII both had significantly increased K_m_ values, consistent with their role in substrate binding. The K_m_ value of R259A was 6000 μM, approximately 22 times that of the wild type, and that of G285D was 4500 μM, approximately 17 times that of the wild type. The k_cat_ values of the two mutants also decreased to only approximately 5% of that of the wild type, suggesting that the two residues affect not only the substrate binding but also the breakage step of chemical bond.

K287A in motif VI was predicted to affect substrate binding. However, the K_m_ value of this mutant was 720 μM, which is only approximately three times that of the wild type. However, the k_cat_ of this mutant was 17.1 s^−1^, which is approximately 1/100 that of the wt. This result suggests that K287 mainly affects k_cat_ rather than K_m_ and may not be involved in substrate binding.

### 2.8. Growth Phenotype of the pApase-Deleted Mutant

*P. yayanosii* CH1 is an obligate piezophilic archaea that is difficult to genetically manipulate. *P. yayanosii* A1 [31], which can grow under atmospheric pressure, was used to knock out the *pApase* gene. The *pApase* was successfully deleted by *hmg*-CoA replacement (Figure S6A,B), but the Δ*pApase* strain did not show an obvious growth phenotype in TRM medium at 95 °C (Figure S6C). Meanwhile, the control strain D1 (insertion of *hmg*-CoA into long gene intergenic regions in the genome) did not result in a growth change.

Whether the deletion of the *pApase* gene inhibits the process of sulfate reduction needs to be tested in a medium that does not contain sulfur-containing substances other than SO_4_. Unfortunately, culture of Strain A1 in defined medium (TRM without yeast extract, peptone, and sulfur but supplied with 0.1 g each of 18 amino acids except Met and Cys and reducing agent FeCl_2_) has been unsuccessful. Therefore, the phenotype of the Δ*pApase* mutant in the context of sulfate assimilation was not confirmed.

## 3. Discussion

### 3.1. PyapApase Converts pAp Efficiently

Bacterial NrnA, RecJ-like protein, archaeal NrnB, and CysQ can convert pAp into 5′-AMP and phosphate with different efficiencies. Among them, the RecJ-like protein *TTHA0118 of T. thermophilus* and CysQ from *E. coli* convert pAp into AMP and phosphate at rates of approximately 160 nmol/μg/min [17] and 33 nmol/μg/min [16], respectively. PyapApase has k_cat_/K_m_ ratio comparable to TTHA0118 and CysQ, but the kinetic parameters are different. The turnover number k_cat_ of PyapApase is 2 μmol/μg/min, more than 10 times that of TTHA0118 and approximately 60 times that of CysQ, while the substrate affinity of CysQ (K_m_ = 1.1 μM) [30] and TTHA0118 (K_m_ = 18 μM) [17] is higher than that of PyapApase (K_m_ = 272 μM). These results suggest that archaeal PyapApase gets the same catalytic efficiency via a largely high turnover number, which is different from bacterial pApases. Compared with TTHA0118, CysQ and PyapApase, other reported pApases show a relatively lower catalytic efficiency. For example, NrnA from *B. subtilis and* Rv2837c from *M. tuberculosis* have turnover numbers of 6 and 1 nmol/μg/min [16,23], respectively. NrnA family protein from *Mycolicibacterium smegmatis* (MSMEG_2630) shows a very low rate*,* approximately 20.9 pmol/μg/min [24], and NrnB nuclease APE_0124 from A. pernix also degrades pAp at a low rate [28], in-di-cating that pAp is not the preferable substrate for MSMEG_2630 and APE_0124.

### 3.2. PyapApase Has High Substrate Specificity

In contrast to PyapApase, bacterial pApase TTHA0118 from *T. thermophilus* can hydrolyze 3-mer ssDNA, and short ssDNA, 3-mer ssDNA, and ssRNA at rates comparable to that of pAp, while cAMP and cGMP can be hydrolyzed at much lower rates [17]. Bacterial Rv2837c from *M. tuberculosis* can hydrolyze c-di-AMP, and pNpN at an efficiency comparable to pAp [23,32]. Bacterial NrnA from *B. subtilis* prefers pAp with thousands of times higher efficiency than nanoRNA [16]. In contrast to the above bacterial pApases, PyapApase is a pApase that is specific to pAp hydrolysis with very high efficiency and does not degrade any other substrates, such as c-di-AMP, c-di-GMP, pApA, pGpG, and nanoRNA. These combined results imply that PYCH_17540 is the intrinsic pApase in *P. yayanosii* cells.

Compared with the wider substrate spectrum of bacterial NrnA [16,23,24], the difference in the substrate-binding cleft may be the main reason why PyapApase cannot hydrolyze larger cyclic dinucleotides and oligonucleotides (Figures S2 and S3). The activity was lost or largely decreased when the conserved residues were mutated (Table 2), suggesting that archaeal pApases have a homologous structure and catalytic mechanism to members of the DHH phosphoesterase superfamily [22].

### 3.3. The Function of PyapApase In Vivo

Sulfate assimilation is an important biological process. It can produce sulfur-containing amino acids and their derivatives and sulfonated products through PAPS and generate the byproduct pAp. The turnover of pAp is very important for normal sulfate assimilation. PyapApase has been identified and may play an important role in sulfate assimilation by turning over pAp in *P. yayanosii*. The gene encoding pApase is generally located in the sulfate assimilation gene cluster, which include genes that encode ATP sulfurylase and APS kinase. In other archaea, a sulfotransferase-encoding gene can be found adjacent to this gene cluster. However, in *P. yayanosii*, sulfotransferase and the core enzyme to transfer sulfite to sulfide (both assimilation and dissimilation) are not identified in the Kyoto Encyclopedia of Genes and Genomes (KEGG). Moreover, a DUF81 protein annotated to be a tauE transporter is also adjacent to this gene cluster. The TauE transporter may transport sulfite anions across the cytoplasmic membrane and is related to sulfotransferase [33].

Although an intact sulfate reduction pathway cannot be found in *P.* *yayanosii*, the possibility that *P. yayanosii* can produce sulfide from sulfate, as nonhomologous genes may possess the same function in sulfate assimilation, cannot be excluded. Even though sulfate cannot be used to produce sulfated metabolites or sulfides, intermediates in sulfate assimilation may also play a role. pAp and PAPS can act as messengers in some organisms, and the function of pAp as a messenger has been well studied in plants and bacteria [11,34,35]. Thus, pAp and PAPS may also be involved in gene regulation as messengers in *P. yayanosii*. Proteome data showed that the expression of PYCH_17550, PYCH_17530, and PYCH_17560 increased under high-pressure conditions, implying that sulfate assimilation may be involved in high-pressure adaptation [36]. Therefore, the significance of sulfate assimilation is still unclear and needs further exploration in archaea. In the future, we will knock out the related genes involved in sulfate assimilation and characterize the mutant phenotypes.

## 4. Materials and Methods

### 4.1. Materials

*E. coli* strain DH5α was used for gene cloning, and the Rosetta2(DE3)pLysS strain was used for recombinant protein expression. The expression vector pET28a was used to express the recombinant proteins. *P. yayanosii* genomic DNA was extracted from *P. yayanosii* cultures in our lab. PrimSTAR Max DNA polymerase was purchased from Takara (Shiga, Japan). Nickel–nitrilotriacetic acid resin was purchased from Bio-Rad (Hercules, CA, USA). Adenosine 3′,5′-bisphosphate (pAp) was purchased from Sigma (St Louis, MO, USA). The Cycle Pure Kit was purchased from Omega (Guangzhou, China). Methanol, formic acid, and ammonium formate were purchased from Aladdin (Shanghai, China) for the HPLC assay.

### 4.2. Construction of Phylogenetic Trees of pApases and Other Members of the DHH Superfamily

As bidirectional best hits of pApase via Basic Local Alignment Search Tool (BLAST), pApase sequence homologs from different archaeal and bacterial groups were obtained from NCBI (http://ncbi.nlm.nih.gov/protein). A phylogenetic tree was constructed based on the amino acid residue sequences of pApase homologs using MEGA version X [29]. Bootstrap analysis based on 1000 replications was used to estimate the confidence level of tree topologies.

### 4.3. Preparation of Recombinant pApase and Its Mutants

The *pApase* (ORF *pych_17540*) gene was amplified from *P. yayanosii* genomic DNA by PCR using specific primers (Supplementary Table S1) and then inserted into the pET28a vectors between the *Nde* I and *Xho* I restriction sites. Subsequently, amino acid substitutions were introduced into PyapApase with a QuikChange^®^ Site-Directed Mutagenesis Kit from Stratagene (Santa Clara, CA, USA) using PrimeSTAR Max DNA polymerase and appropriate primers (Table S1). The wt and mutated *pApase* genes were verified to be correct by sequencing the whole gene sequences.

### 4.4. Protein Expression and Purification

Recombinant plasmids were introduced into the *E. coli* Rosetta2(DE3)pLysS strain to express the recombinant proteins. The clones that contained different recombinant plasmids were cultured in 5 mL of Luria-Bertani (LB) medium at 37 °C and 200 rpm until OD_600_ = 1.5–2. Two milliliters of culture was inoculated into 150 mL of LB medium and further cultured at 37 °C and 200 rpm until OD_600_ = 0.5–0.6. After cooling at 4 °C for 20 min, isopropylthio-β-galactoside (0.15 mM final concentration) was added to the culture to induce the expression of recombinant proteins at 20 °C for 15–17 h. Recombinant proteins were purified via immobilized Ni^2+^ affinity chromatography as follows. The bacterial pellet was resuspended in lysis buffer (20 mM Tris-HCl, pH 8.0, 300 mM NaCl, 5 mM β-mercaptoethanol, 10 mM imidazole, 1 mM phenylmethylsulfonyl fluoride, and 10% glycerol (*v*/*v*)) and then disrupted by sonication. After incubation at 65 °C for 30 min to inactivate most host proteins, the cell extracts were clarified by centrifugation at 12,000× *g* for 30 min at 4 °C. After loading the supernatant onto a Ni^2+^ affinity column pre-equilibrated with lysis buffer, the resin was washed with 100 column volumes of lysis buffer containing 20 mM imidazole. Finally, the bound protein was eluted from the column using elution buffer (20 mM Tris-HCl, pH 8.0, 300 mM NaCl, 5 mM β-mercaptoethanol, 200 mM imidazole, and 10% glycerol (*v*/*v*). After verifying the purity of the elutes by 12% sodium dodecyl sulfate-polyacrylamide gel electrophoresis (SDS-PAGE), the protein preparations were dialyzed against storage buffer consisting of 20 mM Tris-HCl, pH 8.0, 100 mM NaCl, and 50% glycerol (*v*/*v*) at 4 °C and then stored in small aliquots at −20 °C.

### 4.5. Optimization of the Reaction Conditions

The activity of PyapApase toward pAp was first characterized in 20 μL of basic buffer consisting of 20 mM Tris-HCl (pH 8.0), 50 mM NaCl, and 2.0 mM MnCl_2_. pAp (0.2 mM) was incubated with 3.75 nM PyapApase or D12A mutant at 70 °C for 5 min. After incubation, 1 μL of 500 mM EDTA was added to the reaction to inactivate PyapApase. The substrate and product were analyzed by HPLC (Section 4.7). The optimal pH value was determined using different reaction buffers: 20 mM MES-NaOH (pH 5.0–7.00), 20 mM Tris-HCl (pH 7.0–9.0), and 20 mM Gly-NaOH (pH 9.0–11.0). To determine the effect of different divalent metal ions on pApase, each kind of ion, Mg^2+^, Ca^2+^, Ni^2+^, Co^2+^, Cu^2+^, Zn^2+^, and Mn^2+^, at 1 mM was included in the reaction buffer. EDTA (25 mM) was added to the reaction buffer to verify its effect on pApase. The optimal concentration of divalent metal ions (Ni^2+^, Co^2+^, and Mn^2+^) was determined via ranging from 0 to 2 mM. The optimal NaCl concentration was determined by adjusting the NaCl concentration to 15, 50, 100, 200, and 500 mM. Finally, the effect of temperature on enzyme activity was determined at 25, 40, 60, 80, and 90 °C.

### 4.6. Characterization of Enzymatic Activity on Dinucleotide Substrates

Dinucleotide derivatives, including c-di-AMP, c-di-GMP (Sigma, St Louis, MO, USA) pApA, and pGpG (Biolog, Hayward, CA, USA), were used as substrates in the hydrolysis reaction (20 μL) containing 20 mM Tris-HCl (pH 8.0), 50 mM NaCl, 1.0 mM DTT, 1.0 mM MnCl_2_, and 100 µg/mL BSA. PyapApase (1.5 μM) was incubated with 0.075 mM dinucleotide derivatives at 70 °C for 10 min to verify its activity. After incubation, 1 μL of 500 mM EDTA was added to the reaction to inactivate PyapApase.

### 4.7. Analyzing Substrate and Product by HPLC

Subsequently, a C18 analytical column (RP-C18, 4.6 × 250 mm, 5 µm particle size) from Agilent Co. (Lexington, MA, USA) was used to separate the product and substrate on the HPLC system (Agilent). The optimized mobile phase for desorption and separation was a mixture of 90% buffer A (0.137 M ammonium formate and 0.273 M formic acid) (*v*/*v*) and 10% methanol (*v*/*v*), and the flow rate of the mobile phase was kept at 1.0 mL/min. The detection of substrate and product was conducted through absorption at 254 nm with a scanning range of 200–360 nm.

For analysis of the linear and cyclic dinucleotides, the optimized mobile phase for desorption and separation was a mixture of 90% buffer A (30 mM K_2_HPO_4_ and 20 mM KH_2_PO_4_ adjusted to pH 6 by H_3_PO_4_) (*v*/*v*) and 10% methanol (*v*/*v*) [37]. The flow rate of the mobile phase and the detection of substrate and product were the same as for pAp.

### 4.8. Measurement of Enzyme Activity toward ssDNA and ssRNA

For analysis of the activity toward ssDNA and ssRNA substrates, 5′-FAM labeled short nanoRNA (4 nt), longer ssRNA (12 nt), nanoDNA (4 nt), and longer ssDNA (23 nt) at 100 nM each were incubated with 1.5 μM PyapApase in the reaction buffer at 55 °C for 15 min. After incubation, 20 μL of stopping buffer (90% formamide, 100 mM EDTA, 0.25% SDS, and 0.25% bromophenol blue) was added to the reactions to inactivate the enzyme. Then, the substrate and products were separated by 15% 8 M urea-denatured PAGE, and the gels were imaged using a Typhoon 9500 fluorescent scanner (GE Healthcare, Boston, MA, USA). The sequences of ssDNA and ssRNA are listed in Table S3. The archaeal NrnA Ape_0124 was prepared, and its ability to hydrolyze nanoRNA and nanoDNA was analyzed as previously described [28].

### 4.9. Crystallization, Structure Determination, and Refinement

Selenomethionine (SeMet)-labeled PyapApase was expressed using the methionine auxotrophic *E. coli* strain B834 (DE3) in a defined medium and purified similar to native protein. The affinity-purified SeMet-labeled PyapApase and PyapApase mutants of DHH^77–79^AAA and H79A were further purified through a Hiload Superdex 200 column (120 mL, GE Healthcare) using a balance buffer consisting of 25 mM Tris-HCl (pH 8.0) and 150 mM NaCl. The three PyapApases were concentrated to 17 mg/mL for crystallization. After mixing equal volumes of protein and reservoir solution, the hanging drop vapor-diffusion method was used to grow the crystals of PyapApases at 18 °C. The reservoir solution used for SeMet-labeled PyapApase contained 0.2 M sodium acetate, 0.1 M sodium cacodylate pH 6.5, and 30% (*w*/*v*) PEG8000. The reservoir solution used for the PyapApase H79A mutant contained 8.25% PEG3350 (*w*/*v*), 1.65% (*w*/*v*) PEG400, and 0.1 M sodium acetate/acetic acid, pH 5.5. The reservoir solution used for the PyapApase DHH^77–79^AAA mutant consisted of 9.9% (*w*/*v*) PEG1500, 3.3% (*v*/*v*) isopropanol, 0.1 M CaCl_2_, and 0.1 M imidazole/hydrochloric, pH 6.5. Some crystals were harvested after two weeks and then flash-frozen in liquid nitrogen for diffraction and data collection. All X-ray diffraction data sets were collected at 100 K at BL17U1 of the Shanghai Synchrotron Radiation Facility. Indexing, integration, scaling, and merging of the diffraction data were performed by using the HKL2000 program suites [38,39].

The structure of SeMet-labeled apo-PyapApase was determined using the single-wavelength anomalous dispersion (SAD) method. The structures of the two mutants PyapApases were determined by molecular replacement. The primary structure was solved using the autoSHARP pipeline [40]. Then, maximum likelihood-based refinement of the atomic positions and temperature factors was performed using Phenix [41]. The atomic model was fit using the program Coot 0.94 [42]. The stereochemical quality of the final model was assessed using MolProbity 4.2 [43]. The data collection statistics and the refinement statistics of the PyapApase structures are shown in Table 1. Figures were prepared with PyMOL [Schrodinger LLC (2012) The PyMOL Molecular Graphics System, version 1.5.0.3].

### 4.10. Enzyme Kinetics

K_m_ and k_cat_ were calculated by double reciprocal plotting using the initial reaction rates at various substrate concentrations (0.1, 0.2, 0.5, 1.0, and 2.0 mM). The kinetic parameters were determined in the presence of wt PyapApase (1.5 nM) and mutated enzymes (225 nM D12A, 75 nM R259A, 30 nM G285D, and 30 nM K287A). All reactions (20 μL) were performed in buffer consisting of 20 mM Tris-HCl (pH 8.0), 50 mM NaCl, 1.0 mM DTT, 1.0 mM MnCl_2_, and 100 µg/mL BSA at 70 °C. Then, 1 μL of 500 mM EDTA was added to the reaction to inactivate PyapApase, followed by HPLC analysis of the product. The product AMP was quantified to calculate the initial rates. All data are the means of three independent experiments.

### 4.11. Deletion of pApase in P. yayanosii A1

*P. yayanosii* A1, a mutant strain derived from the obligate piezophilic strain *P. yayanosii* CH1 that can grow under atmospheric pressure [31,44,45], was used to delete the ORF *pych_17540* in the genome. *P. yayanosii* A1 was cultured in serum bottles or tubes plugged with rubber plugs and sealed with aluminum caps under anaerobic conditions in TRM medium at 95 °C in an incubator. Anaerobic conditions were guaranteed by more than three cycles of vacuuming and nitrogen filling and then supplemented with reducing agent Na_2_S·9H_2_O or polysulfide solution. The liquid TRM medium was supplemented with 1% (*w*/*v*) sulfur powder and 1% (*v*/*v*) Na_2_S·9H_2_O (pH 7.0, 10% (*w*/*v*)), and the solid TRM medium was supplemented with 0.1% polysulfide solution (containing 10 g of Na_2_S·9H_2_O, 15 mL of ddH_2_O, and 3 g of sulfur powder) [46]. Simvastatin was added to TRM medium at a final concentration of 10 μM for positive selection in solid medium and positive selection culture in liquid medium.

To delete the *pApase* gene, pApase was replaced with the frame of the *hmg*-CoA gene fragment. The linearized fragments (upstream + *hmg*-CoA + downstream) for recombination were generated by PCR using the primers in Table S2 and were introduced into strain A1 using the CaCl_2_ method [44]. After transformation, the mixture containing transformed cells was spread on solid TRM supplemented with liquid polysulfide and 10 μM simvastatin. Cells were cultured at 95 °C for approximately 24 h until colonies were observed. The colonies were picked and cultured in liquid medium for isolation of positive clones. PCR amplification was performed using primers P1 and P6′ (Table S2) to determine whether the *pApase* gene was replaced by the frame of *hmg*-CoA. To construct a control and evaluate the effect of the frame of *hmg*-CoA, the gene was inserted between *pych_00780* and *pych_00790* using a set of primers (Table S2), as described for replacing the *pApase* gene with the frame of *hmg*-CoA.

The above mutant and wild-type strains were grown in TRM liquid medium and monitored by cell counting using a Thomas chamber and light microscopy at a magnification of 40× [44,47]. Each cell counting experiment was repeated in three biological replicates.

## Figures and Tables

**Table 1:** Data collection and refinement statistics.

Parameter	Se-WT	DHH^77–79^AAA	H79A
**Data collection**			
Wavelength (Å)	0.9792	0.9792	0.9792
Space group	P2_1_	P12_1_1	P12_1_1
Cell dimensions			
a, b, c (Å)	58.6, 67.1, 60.3	61.5, 149.4, 75.9	59.26, 72.3, 76.21
β (°)***b***	112.4	90, 99.3, 90	90, 101.73, 90
Resolution (Å)	50–2.10 (2.14–2.10)	34.45–2.69 (2.76–2.69)	29–2.05 (2.13–2.05)
No. reflections	37,590	37,365	38,863
***R***_merge_^b^ (%) ^a^	8.5 (70.4)	9 (157.4)	13.2 (124.5)
Mean I/σ (I) ^a^	23.5 (10.2)	18.2 (1.9)	14.1 (3.7)
Completeness (%) ^a^	100 (100)	99.46 (99.6)	98.36 (96.98)
Redundancy ^a^	7.5 (3.1)	6.8 (5.7)	6.7 (6.0)
**Refinement**			
***R***_work_/*R*_free_ (%) ^c^	17.37/21.00	22.63/27.38	19.32/22.96
No. atoms			
Protein	3702	9832	4928
Water	178	15	241
Ligand	2	/	/
R.M.S. Deviation			
Bond lengths (Å)	0.002	0.007	0.022
Bond angles (°)	0.473	1.15	1.08
Ramachandran plot (%)			
Favored	98.29	94.57	96.58
Allowed	1.28	5.03	2.93
Outliers	0.43	0.41	0.49

^a^ The values in parentheses are for the outermost shell. ^b^ R_merge_ = ∑*_hkl_*∑*_i_*|I_i_(hkl) − < I(hkl) > |/∑*_hkl_*∑*_i_*|I_i_(hkl), where < I(hkl) > is the mean intensity of a set of equivalent reflections. ^c^ *R_work_* = ∑*_hkl_*||*F_obs_*| − |*F_calc_*||/∑*_hkl_*_|*Fobs*|_, where *F_obs_* and *F_calc_* are observed and calculated structure factors, respectively. *R*_free_, calculated the same as *R*_work_ but from a test set containing 5% of data excluded from the refinement calculation.

**Table 2:** Kinetic parameters of wt and mutant PyapApases.

Protein	K_m_	k_cat_	k_cat_/K_m_
WT	272 ± 12.6 μM	1259 ± 94.1 s^−1^	4.63 × 10^3^ M^−1^s^−1^
D12A	790 ± 47.7 μM	1.7 ± 0.3 s^−1^	2.16 × 10^3^ M^−1^s^−1^
R259A	6000 ± 753.4 μM	61.57 ± 7.1 s^−1^	1.03 × 10^3^ M^−1^s^−1^
G285D	4500 ± 607.7 μM	71.04 ± 9.6 s^−1^	1.58 × 10^3^ M^−1^s^−1^
K287A	720 ± 33.1 μM	17.10 ± 2.1 s^−1^	2.38 × 10^4^ M^−1^s^−1^

K_m_ and k_cat_ were calculated by double reciprocal plotting using the initial reaction rates at various substrate concentrations (0.1, 0.2, 0.5, 1.0, and 2.0 mM). The kinetic parameters were determined in the presence of wt PyapApase (1.5 nM) and mutated enzymes (225 nM D12A, 75 nM R259A, 30 nM G285D, and 30 nM K287A). The product AMP was quantified to calculate the initial rates. All data are the means of three independent experiments.

## Data Availability

This data supporting crystal structures have been uploaded to PDB.

## Supplementary Materials

The following are available online at https://www.mdpi.com/article/10.3390/ijms22137159/s1.
