# Supplementary material for: Structure and Function of Piezophilic Hyperthermophilic Pyrococcus yayanosii pApase
Source: Int J Mol Sci. 2021 Jul 2;22(13):7159. doi: 10.3390/ijms22137159 (PMC8268124; doi:10.3390/ijms22137159)
Supplement: Supplementary file 1 [file ijms-22-07159-s001.zip › ijms-1222881-supplementary.pdf]

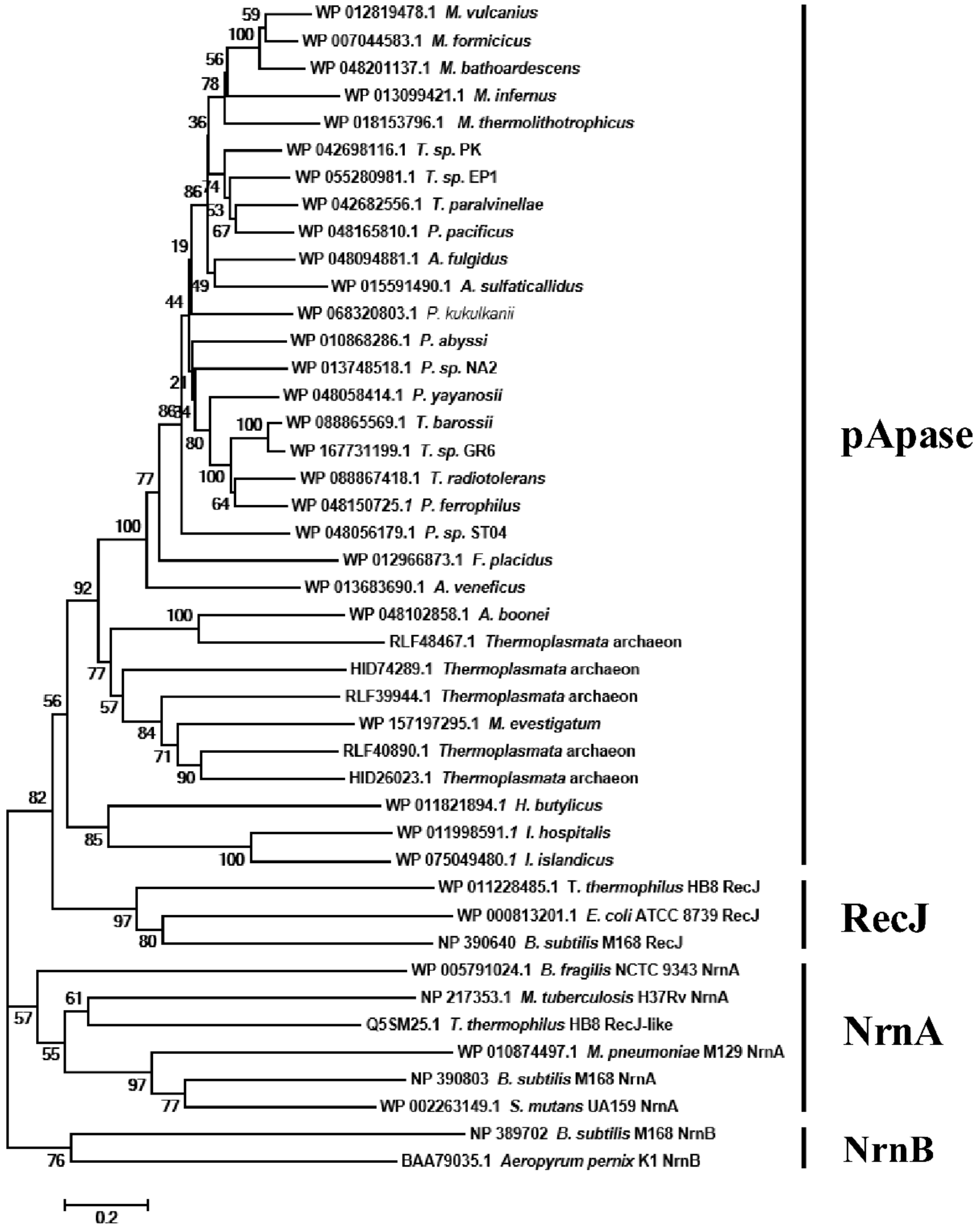

Figure S1. Phylogenetic tree for selected members of the DHH superfamily. Sequence homologs of the pApase, RecJ, NrnA, and NrnB subfamilies were obtained from NCBI (http://ncbi.nlm.nih.gov/protein) and used to construct the phylogenetic tree. Genetic location and neighboring genes were identified from GenBank. Bootstrap values were expressed as a percentage of 1000 replicates.

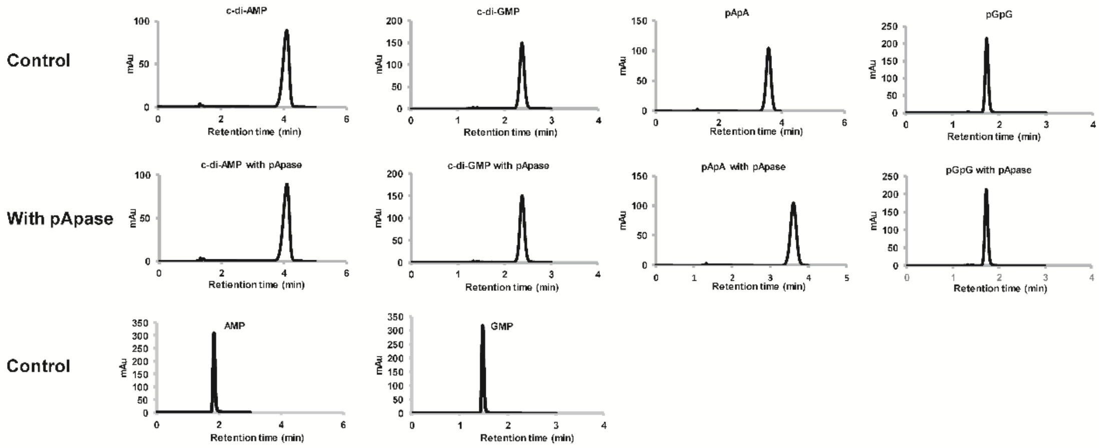

Figure S2. Linear and cyclic adenosine and guanosine dinucleotides are not substrates of PyapApase. Linear and cyclic adenosine and guanosine dinucleotides (0.075 mM) were incubated with 1.5 μM of PyapApase in reaction buffer. Each row is the negative control (substrates), pApase-treated substrates, and positive control (products), from top to bottom.

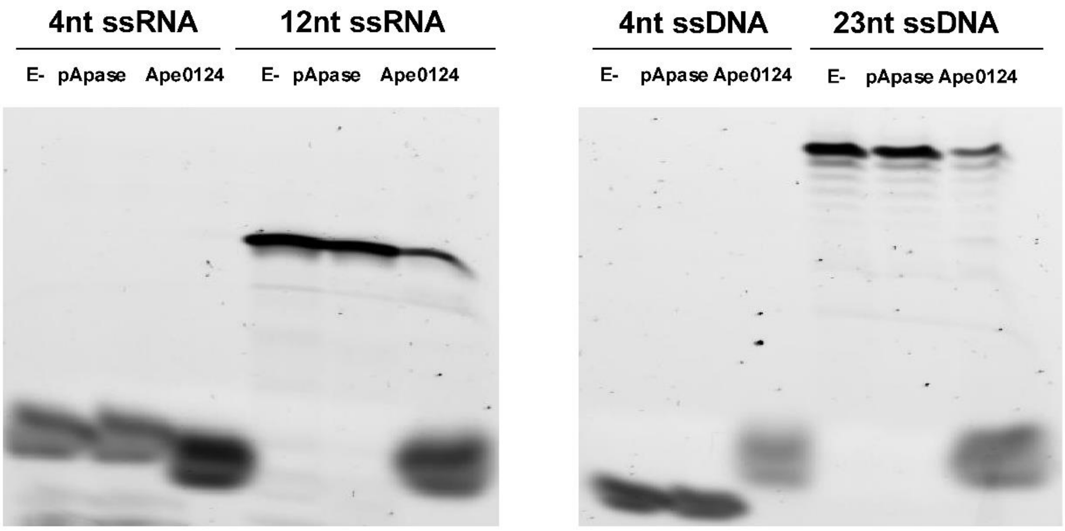

Figure S3. PyapApase does not hydrolyze single-stranded RNA and DNA. Short nanoRNA (4 nt), longer ssRNA (12 nt), nanoDNA (4 nt), and longer ssDNA (23 nt) were incubated with PyapApase in reaction buffer at 55 °C for 15 min. The reactions were separated by 15 % 8 M urea-denatured PAGE, and bands in the gels were imaged using a Typhoon 9500 fluorescent scanner (GE Healthcare). The sequences of ssDNA and ssRNA are listed in Table S3. The archaeal NrnA Ape_0124 was prepared, and its ability to hydrolyze nanoRNA and nanoDNA was analyzed as previously described [28].

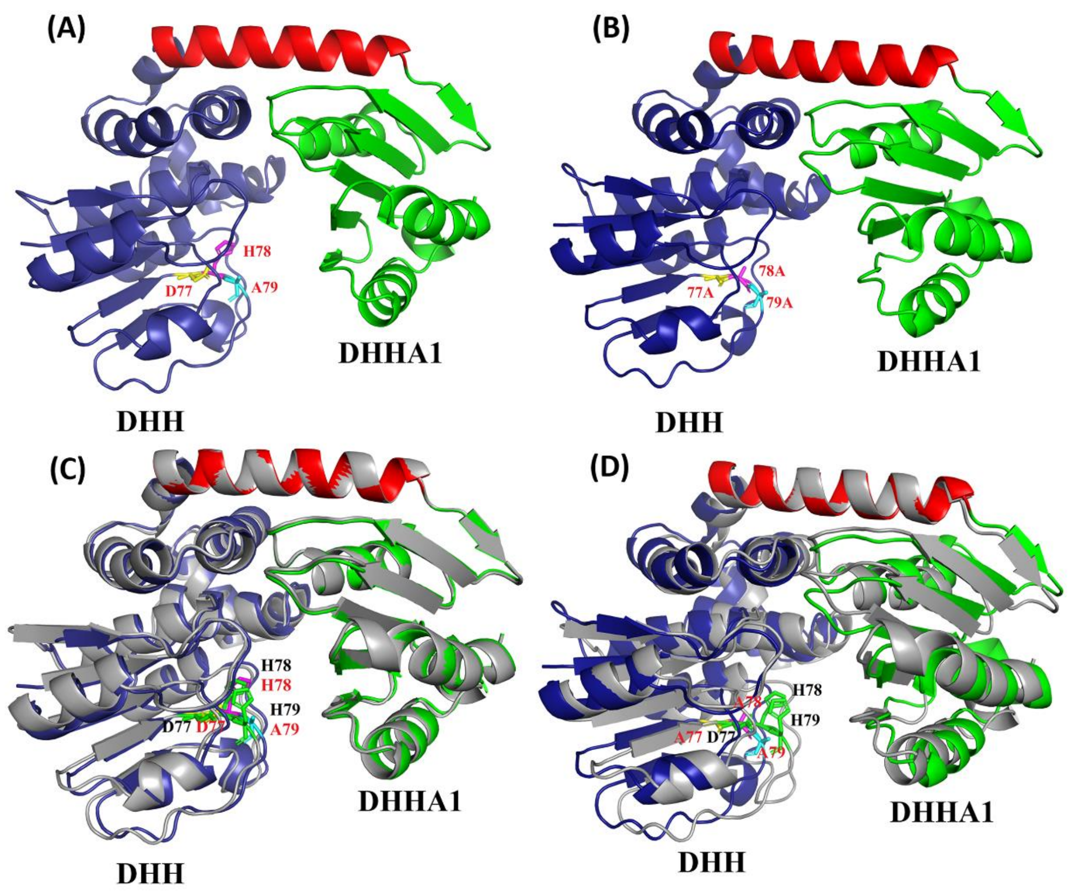

Figure S4. Structural comparison of H^{79}A and DHH$^{77-79}$AAA mutants and wt PyapApase. The structure of H^{79}A (A) and DHH$^{77-79}$AAA (B). The conserved DHH residues in motif III are thought to coordinate the divalent ions and were mutated to one alanine (H^{79}A) or three alanines (DHH$^{77-79}$AAA). The three residues of motif III are marked and depicted in stick-and-ball form. (C and D) The structures of H^{79}A and DHH$^{77-79}$AAA mutants were compared with wt PyapApase. wt PyapApase is shown in gray, and the DHH residues are shown in black. The two mutants are shown in the same color as in panels A and B, and the residues in the mutated DHH motif are shown in red.

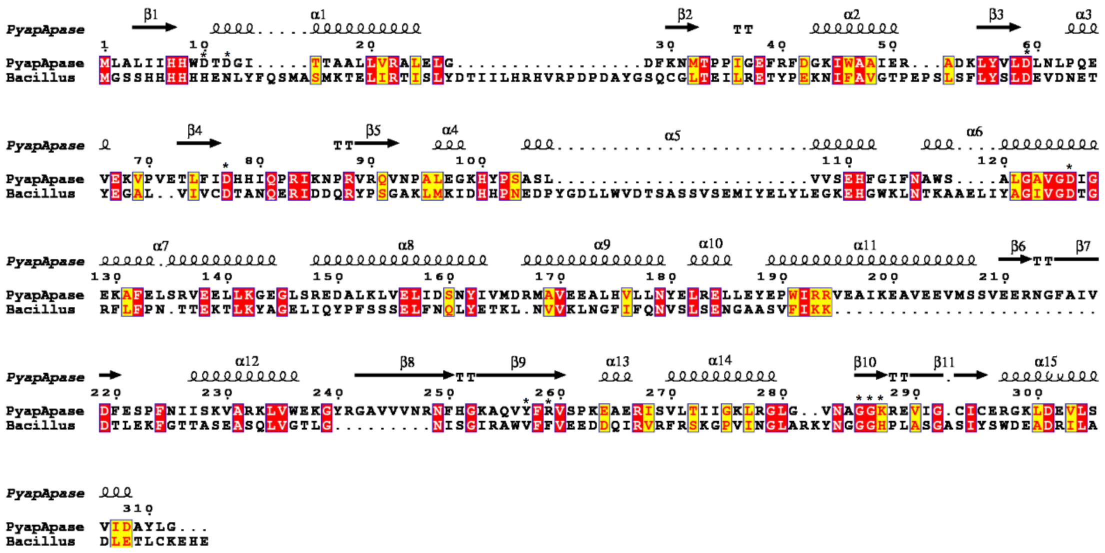

Figure S5. Multiple sequence alignment of PyapApase and BsNrnA. The secondary structure element of PyapApase is represented at the top. The horizontal helixes indicate α-helixes, and arrows indicate β-strands. Completely conserved residues between PyapApase and BsNrnA are boxed and shaded in red. DDH domain residues that bind metal ions and DHHA1 domain residues that bind single nucleotides are marked with stars. The alignment was carried out using the multiple sequence alignment program ClustalW.

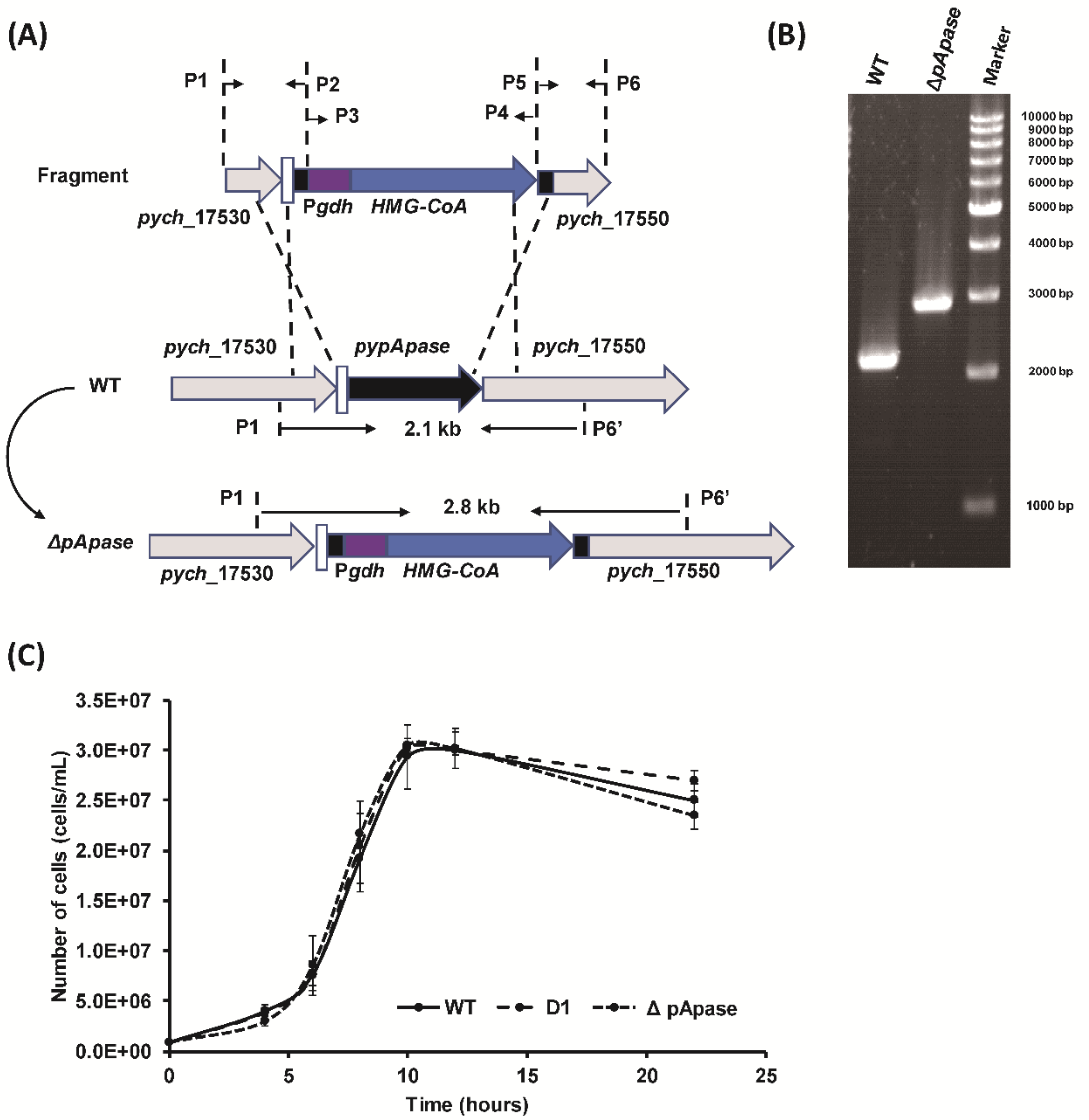

Figure S6. Deletion of the pApase gene does not affect cell growth. (A) Δ*pApase* was constructed by homologous recombination using a linear DNA fragment that contains an upstream region of the *pApase* gene, including 100 bp of *pApase* (amplified by P1 and P2), a positive selection marker gene consisting of the *gdh* promoter (shown in purple line) and the *HMG-CoA* encoding sequence (shown by a blue arrow, amplified by P3 and P4), and a downstream region of the *pApase gene*, including 100 bp of *pApase*. (B) Δ*pApase* was verified by PCR amplification using P1 and P6' (downstream of P6, shown in panel A). The pApase gene was replaced with the selection marker gene in the Δ*pApase* mutant. (C) Growth curve of Δ*pApase*, strain D1 harboring a selection marker gene, and wild-type strain in liquid TRM medium at 95 °C.

Table S1. Sequences of primers for constructing wt and site-directed mutated pApase expression vectors

Primer	Sequence (5'-3') [a]
PyapApase-F	CCCCCCGCTAGCATGTTGGCCCTCATTATCCATCA
PyapApase-R	CCCCCCCTCGAGTCACCCCAGGTAAGCATCGATTA
D10A F	TATCCATCATTGGGcCACGGACGGCATAAC
D10A R	GTTATGCCGTCCGTGgCCCAATGATGGATA
D12A F	CATTGGGACACGGcCGGCATAACAAC
D12A R	GTTGTTATGCCGgCCGTGTCCCAATG
D59A F	CTTTACGTCCTCGcCTTAAATCTCCCC
D59A R	GGGGAGATTTAAGgCGAGGACGTAAAG
D77A F	GAAACGCTCTTCATAGcCCACCACATCCAG
D77A R	CTGGATGTGGTGGgCTATGAAGAGCGTTTC
H78A F	GCTCTTCATAGACgcCCACATCCAGCCG
H78A R	CGGCTGGATGTGGgcGTCTATGAAGAGC
H79A F	CTTCATAGACCACgcCATCCAGCCGAGG
H79A R	CCTCGGCTGGATGgcGTGGTCTATGAAG
77DHH79AAA F	AAACGCTCTTCATAGcCgcCgcCATCCAGCCGAGG
77DHH79AAA R	CCTCGGCTGGATGgcGgcGgCTATGAAGAGCGTTT
D126A F	GGTGCTGTGGGTGcTATAGGTGAGAAAG
D126A R	CTTTCTCACCTATAgCACCCACAGCACC
Y257A F	GAAAGGCTCAGGTTgcCTTCAGAGTCTCAC
Y257A R	GTGAGACTCTGAAGgcAACCTGAGCCTTTC
R259A F	CTCAGGTTTACTTCgcAGTCTCACCGAAG
R259A R	CTTCGGTGAGACTgcGAAGTAAACCTGAG
G285D F	GGGTGAACGCGGaCGGTAAGAGGGAG
G285D R	CTCCCTCTTACCGtCCGCGTTCACCC
G286D F	GAACGCGGGCGaTAAGAGGGAGGTAAT
G286D R	ATTACCTCCCTCTTAtCGCCCGCGTTC
K287A F	CGCGGGCGGTgcGAGGGAGGTAATTG
K287A R	CAATTACCTCCCTCgcACCGCCCGCG

[a] The red bases denote the restriction site or site-mutated bases.

Table S2. Sequences of primers for constructing mutant strains of *pApase* and D1

Strains	Primer	Sequence [a] (5'-3')
	P1	TGGAGCAACGCACTTCATCGT
	P2	**TCCATTTTCAAGTGTCATGTTC**TTGAAATCCCC
	P3	**GAACATGACACTTGAAAATGGA**GTGAGCTGAGT
pApase	P4	**CCTCCCTCTTTCATCTCCCA**AGCATTTTATGA
	P5	**TGGGAGATGAAAGAGGGAGG**TAATTGGTTGTA
	P6	ACCAGCCATTTATCTTCTTTG
	P6'	ACAGCCGTCTCATCCAAGTC
	D1 P1	TACCTCAGAAACGTCTTCATAG
	D1 P2	**ACTCCATTTTCAAGTTTGTAATTTTT**CAGGATCTTGA
	D1 P3	**AAAAATTACAAACTTGAAAATGGAGT**GAGCTGAGT
D1	D1 P4	**CACTCCAGGGTCATCTCCCA**AGCATTTTATGA
	D1 P5	**TGGGAGATGACCCTGGAGTG**AAGACTGAATAC
	D1 P6	CTCACTTTTCGTTTCTGAAGCC

[a] Overlap regions are shown in bold letters.

Table S3. Sequences of ssDNA and ssRNA substrates used in Figure S3

Substrates	Sequence (5’-3’) [a]
4 nt ssRNA	*acgu
12 nt ssRNA	*cggagaugacgg
4 nt ssDNA	*CGAT
23 nt ssDNA	*TCCGATAGCCAGATATCTTGACA

[a] Asterisks denote the fluorescein (6-FAM) moiety at the 5’-end. Lowercase letters represent RNA, and uppercase letters represent DNA.
